# Single-Step Extrusion Printing of Microgrooved Annulus Fibrosus Scaffolds via Patterned Nozzles

**DOI:** 10.3390/jfb17030140

**Published:** 2026-03-11

**Authors:** Nadine Kluser, Gion Ursin Alig, Christoph Sprecher, Xavier Woods, Sibylle Grad, Mauro Alini, Sonja Häckel, Christoph E. Albers, David Eglin, Rajkishen Narayanan, Andrea J. Vernengo

**Affiliations:** 1 AO Research Institute Davos, 7270 Davos Platz, Switzerland; 2 Federal Institute of Technology, ETH Zurich, 8092 Zurich, Switzerland; 3 Department of Chemical Engineering, Rowan University, Glassboro, NJ 08028, USA; 4 Department of Orthopaedic Surgery and Traumatology, Inselspital, Bern University Hospital, University of Bern, Freiburgstrasse 18, 3010 Bern, Switzerland; 5 Department of Spine Surgery, Sonnenhof Spital, Salvisbergstrasse 4, 3006 Bern, Switzerland; 6 Mines Saint-Etienne, Universite Jean Monnet, INSERM, UMR 1059 Sainbiose, 42023 Saint-Etienne, France; 7 Department of Orthopaedic Surgery, Bone and Joint Institute, Cooper University Health Care, Camden, NJ 08103, USA

**Keywords:** annulus fibrosus, intervertebral disc, 3D printing, extrusion-based biomanufacturing, microgrooves, contact guidance, cell alignment, spatial organization, angle-ply architecture, PCL scaffolds, mesenchymal stem cells

## Abstract

Intervertebral disk pathology, including disk herniation and degeneration, is a major contributor to chronic low back pain, and when conservative treatment fails, surgical management often involves discectomy-based procedures that leave residual annulus fibrosus (AF) defects associated with reherniation and progressive degeneration. These limitations have motivated interest in regenerative strategies using biomaterial scaffolds; however, reproducing the hierarchical, angle-ply architecture of the AF remains challenging. Here, we present a single-step extrusion-based 3D-printing approach to fabricate polycaprolactone (PCL) scaffolds with aligned microscale surface grooves that promote AF-like organization. Patterned nozzles with circumferential peaks generated uniaxial concave microgrooves (10–17 µm wide) directly during printing, enabling formation of multilamellar angle-ply constructs. Human bone marrow-derived mesenchymal stem cells cultured on patterned scaffolds aligned longitudinally within concave grooves, forming end-to-end arrays that guided extracellular matrix deposition. Gene expression analysis showed that topographical cues governed cellular organization without significantly altering gene expression profiles, while TGF-β3 supplementation upregulated outer AF-associated markers, including COL1, COL12, SFRP2, MKX, MCAM, and SCX. TAGLN expression increased specifically on patterned scaffolds in the absence of TGF-β3, indicating an association between microgroove-guided cellular organization and TAGLN expression, warranting further investigation into potential tension-related mechanisms. This novel single-step extrusion-printing approach leverages custom nozzle geometry to impart concave microgrooves, facilitating scalable fabrication of multilamellar angle-ply scaffolds that induce aligned cellular organization and support potential applications in annulus fibrosus repair, as well as mechanobiological studies of anisotropic musculoskeletal tissues.

## 1. Introduction

Low back pain (LBP) represents a major global health and socioeconomic burden, affecting approximately 568 million individuals worldwide [1] and remaining a leading cause of disability [2,3]. Among the structural causes of LBP, intervertebral disk (IVD) pathology, particularly disk herniation associated with annulus fibrosus (AF) disruption, is a major contributor [4,5,6]. Disk herniation frequently presents with radiculopathy and is a leading indication for surgical intervention when conservative management fails [7,8].

The intervertebral disk is a fibrocartilaginous structure that enables load bearing and spinal mobility. It consists of a central gelatinous nucleus pulposus (NP) surrounded by the annulus fibrosus (AF), a multilamellar tissue composed of concentric collagen lamellae arranged in an angle-ply architecture [9,10,11]. This organized architecture provides tensile reinforcement and maintains disk integrity [12].

When the AF is disrupted, the NP extrudes or migrates, causing disk herniation, neural compression, and pain [13,14]. Standard surgical treatment (limited diskectomy, sequestrectomy, or nucleotomy [15,16,17,18,19]) removes the herniated material and reliably relieves radiculopathy [20,21,22]. However, these procedures leave an unrepaired AF defect. This defect is strongly associated with reherniation, persistent diskogenic back pain, accelerated disc degeneration [23,24,25,26], loss of disc height, and long-term biomechanical instability [27,28,29].

To address this, several annulus fibrosus closure devices have been developed to mechanically seal the post-discectomy defect and reduce reherniation risk [30,31,32]. While devices such as Barricaid have demonstrated partial success in selected patient populations [31], limitations related to implant integration, mechanical mismatch, device migration, and lack of biological repair remain significant concerns [33,34,35].

These limitations highlight a persistent unmet clinical need for AF repair strategies that restore both the mechanical function and biological integrity of the annulus fibrosus, motivating the development of tissue-engineered AF scaffolds. A key advancement in the field of AF tissue engineering is the understanding that aligned topographical cues can direct cell elongation, which in turn is critical for promoting AF-like cellular phenotype and organized tissue formation. This process, termed contact guidance, highlights the important role of scaffold architecture in regulating cellular attachment, proliferation, and differentiation [36,37]. Early foundational studies, such as that by Nerurkar et al. [38] and others [37,39,40,41,42,43,44,], demonstrated that fibrous substrates promote cell elongation and anisotropic matrix deposition, resulting in engineered tissues more closely replicating the structure and function of the native AF.

Numerous scaffold fabrication strategies have been explored to reproduce the aligned, multilamellar architecture of the AF [38,45,46,47], though many are associated with inherent limitations. Electrospinning, while capable of producing aligned nanofibers that guide cellular growth in anisotropic tissues due to its ability to mimic ECM topography, often requires cumbersome post-processing steps such as folding, rolling [37,48], stacking, or fusing layers [49,50,51,52,53], to recreate the multilamellar, angle-ply architecture characteristic of the native AF. These manual assembly steps limit scalability and introduce variability.

In this context, extrusion-based 3D printing offers an accessible approach for fabricating scaffolds with precisely controlled macroscale geometries. This layer-by-layer process enables direct construction of complex, three-dimensional architectures that approximate the angle-ply organization of the AF [39,54,55,56]. Furthermore, the use of embedding mediums in 3D printing improves print fidelity [57] and supports the fabrication of complex geometries with unsupported overhangs [39]. This enables continuous filament deposition with controlled spacing and curvature in cylindrical AF-like constructs [58].

However, traditional extrusion-based printing generally produces relatively coarse struts, typically 150–400 µm in diameter [59,60], which exceed the length scale of individual cells and focal adhesions and therefore provide limited direct topographical guidance for cellular organization [60,61,62]. To overcome this limitation, several strategies have been developed to induce cell alignment during bioprinting—for example, systems that incorporate PVA fibrillation and leaching [63] or shear-induced alignment of molecular chains [64]. While these methods can effectively promote anisotropy, they rely on narrow rheological windows and multi-stage fabrication schemes that limit scalability, repeatability, and versatility.

Hybrid approaches that combine electrospinning with 3D printing have also been explored, such as depositing electrospun nanofibers between 3D-printed PCL filaments to provide additional topographic cues [54,65]. However, these constructs are inherently complex, and uniform alignment across the scaffold remains difficult to achieve. Fiber orientation tends to be strongest along or between the printed filaments, while regions farther from the strut interfaces display more variable or branched alignment [65,66]. In addition, many of these methods still require manual assembly, such as rolling 2D electrospun mats into 3D geometries [54,56], further limiting scalability and ease of fabrication.

Collectively, the absence of integrated microscale topographical guidance within conventional single-step extrusion 3D-printing methods continues to pose a challenge for advancing functional AF repair and other tissue-engineering strategies that rely on aligned cellular architectures. Here, we address this challenge with a novel single-step extrusion-printing method using patterned nozzles to directly generate concave microgrooves (10–17 µm) on PCL scaffolds. This innovation integrates microscale surface topography with multilamellar angle-ply architecture during fabrication. In this study, we characterized the microscale features generated by these nozzles and examined how the resulting surface topography influenced bone marrow-derived mesenchymal stem cell (MSC) alignment, phenotypic marker expression, and ECM production—key indicators of AF-like tissue formation.

## 2. Materials and Methods

All chemicals and PCL (Mw = 45 kDa) were purchased from Sigma-Aldrich (St. Louis, MO, USA) if not otherwise stated. TaqMan^TM^ reagents and primers for real-time polymerase chain reaction (PCR) were purchased from Applied Biosystems by Thermo Fisher (Waltham, MA, USA). Cell culture reagents alpha-minimum essential medium (α-MEM), penicillin/streptomycin (Pen/Strep), trypsin, high glucose dulbecco’s modified eagle’s medium (HG-DMEM) and non-essential amino acids were purchased from Gibco (Carlsbad, CA, USA). Fetal bovine serum (FBS), Insulin–Transferrin–Selenium (ITS^TM^ Premix) and ultra-low attachment 24-well plates were purchased from Corning (Corning Incorporated, New York, NY, USA).

### 2.1. Fabrication of 3D-Printed Constructs

3D-printed constructs were fabricated by melt extrusion using a 3D Discovery printer (RegenHu Ltd., Villaz-St-Pierre, Switzerland). PCL pellets were heated to 75 °C and extruded with three different custom-manufactured nozzles: a round nozzle, with inner diameter of 300 µm, and two patterned nozzles, with inner diameters of 300 µm and periodic circumferential peaks 60 or 120 µm high, respectively (Supplementary Figure S1). Molten PCL was extruded through the nozzles at a printing pressure of 1 bar. PCL flowrate (revolutions per meter) was chosen proportionally to nozzle cross-sectional area with 16, 21 and 30 revs/m for the round, and the nozzle with 60 and 120 µm peak height, respectively. A printing speed of 6 mm/s was used for printing the scaffolds in order to achieve high print fidelity, as higher nozzle translation speed was found to result in sagging of the struts and poor conservation of the topography (Supplementary Figure S2). Scaffolds with an angle-ply lattice architecture were designed in BioCAD software (RegenHu Ltd., Villaz-St-Pierre, Switzerland, version1.1-12). The rectangular constructs (10 mm length × 5 mm width) were printed in 4 layers and with filaments oriented at angles of ±30°. Layer thickness depended on the nozzle used, with 0.3 mm for the round nozzle and 0.5 mm for the patterned nozzles. The increased layer height used for patterned nozzles was selected to accommodate the larger effective filament diameter arising from the circumferential peak geometry and to ensure consistent filament stacking and topography preservation across layers. Printing fidelity, filament regularity, and absence of defects were previously validated for this exact nozzle/platform by uniaxial tensile testing and SEM [67], confirming uniform orientation and mechanically competent struts.

### 2.2. 3D-Printed Scaffold Characterization

#### 2.2.1. Light Microscopy

Samples were soaked in liquid nitrogen before cutting perpendicular to the printing direction. A Zeiss light microscope (Carl Zeiss Microscopy GmbH, Jena, Germany) was used to image the cross section and top view of the 3D-printed PCL constructs and peak height was measured (*n* ≥ 29) with AxioVision4 (version 4.9.1.0, Carl Zeiss Microscopy).

#### 2.2.2. Scanning Electron Microscopy

Scanning electron microscopy (SEM) was performed to assess scaffold surface topography. The PCL scaffolds were mounted on an aluminum stub (diameter 12.5 mm), coated with 10 nm Au/Pd (80/20 wt %) and imaged in SEM (Hitachi S-4700 II, Hitachi, Tokyo, Japan). Filament diameter (diameter of outer circumference) and groove width (*n* ≥ 15) were measured from SEM micrographs with ImageJ (version 1.52, National Institutes of Health, Bethesda, MD, USA).

#### 2.2.3. Wettability

**Figure 2:**
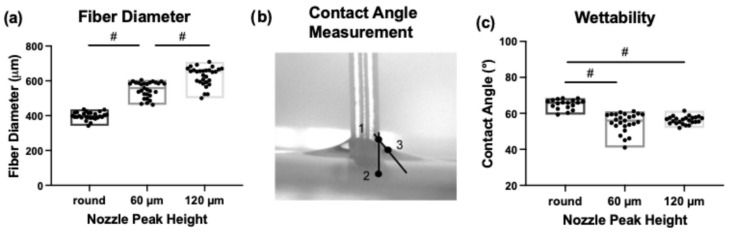
Results of scaffold characterization show nozzle-dependent differences. (**a**) Quantification from SEM images indicated an increase in filament diameter with topographical features. (# *p*-value < 0.05). (**b**) Contact angle measurement was performed by using three predefined points: (1) contact point between water and material, (2) point on the PCL filament and (3) point at the steepest slope on the water meniscus curvature. (**c**) Contact angle measurements indicated decreases in contact angle, or increases in wettability, with larger topographical features. (# *p*-value < 0.05).

Wettability was determined using contact angle measurement. A typical droplet size in contact angle measurement on PCL ranges from 2 to 10 µL [68,69,70,71,72,73,74,75,76,77], which would cover the whole surface of a 3D-printed filament. Therefore, extruded PCL filaments were immersed in Milli-Q^®^ water and pictures were continuously taken while raising the filaments slowly from the water with DinoCapture Camera (AnMo Electronic Corporation, Taipei, Taiwan). Contact angle was measured on the pictures (*n* ≥ 18) with AxioVision4 (version 4.9.1.0, Carl Zeiss Microscopy, Jena, Germany). Three points were defined to measure contact angle at the water material interface: (1) contact point between water and filament, (2) point on the PCL filament, and (3) point at the steepest slope on the water meniscus curvature (Figure 2b).

### 2.3. Cell Culture

Bone marrow-derived mesenchymal stem cells (BM-MSCs) from three donors (one male (donor 1), age 85, and two females (donors 2 and 3), ages 33 and 55) undergoing spinal surgery were isolated and cryopreserved at a density of 2 × 10^6^ cells per ml. General Consent which also covers anonymization of health-related data and biological material was obtained from all donors. After thawing (passage 1), cells were suspended in growth medium containing α-MEM, 10% FBS, 1% Pen/Strep and 5 ng/mL basic fibroblast growth factor (b-FGF, Fitzgerald Industries, Acton, MA, USA) and were seeded in T300 tissue culture flasks (TPP, Trasadingen, Switzerland). Non-adherent cells were removed after 24 h and medium was exchanged three times per week. Cells were passaged upon reaching 80% confluency.

Prior to seeding with cells, 3D-printed PCL scaffolds were sterilized in ethylene oxide (EtO), placed in ultra-low attachment 24-well plates and pre-wetted in growth medium for 24 h. Then, third-passage BM-MSCs were trypsinized (0.5% Trypsin EDTA in PBS) and suspended in growth medium at a concentration of 5 × 10^5^ cells/mL. A total of 2.5 × 10^5^ cells were seeded per scaffold and allowed to attach for 48 h prior to removing non-adherent cells and changing to differentiation medium. The samples were cultured in AF differentiation medium containing high glucose DMEM (4.5 μg/mL D-Glucose), sodium pyruvate (0.11 μg/mL) and sodium bicarbonate (3.7 μg/mL) with 1% Pen/Strep, L-ascorbic acid 2 phosphate (50 μg/mL), 1% ITS, non-essential amino acids (5 mg/mL), dexamethasone (0.04 ng/mL) and either 0 (control) or 10 ng/mL recombinant TGF-β_3_ [78] (PreproTech, Rocky Hill, CT, USA). Medium was exchanged three times per week and samples were evaluated after 48 h (week 0) and 4 weeks of culturing in AF differentiation medium.

### 2.4. Cell Morphology and Alignment

#### 2.4.1. Cytoskeletal and Focal-Adhesion Staining

To evaluate cellular morphology and alignment, samples were fixed using paraformaldehyde (4% in PBS) for 20 min at room temperature and washed three times with PBS. Samples were incubated with an actin cytoskeleton and focal-adhesion staining kit, containing TRITC-conjugated phalloidin, anti-vinculin and DAPI following the manufacturer’s protocol (Millicore, Sigma). Briefly, cells were permeabilized with 0.1% Triton X-100 in PBS for 5 min and incubated with anti-vinculin at a dilution of 1:200. AlexaFluor488 goat anti-mouse secondary antibody (2 mg/mL, Invitrogen, Thermo Fisher Scientific) was double stained with TRITC-conjugated phalloidin at a dilution of 1:200 and incubated for 60 min at room temperature. DAPI staining (1:1000) was performed subsequently and samples were washed with wash buffer (1× PBS containing 0.05% Tween-20) prior to further analysis.

#### 2.4.2. Image Acquisition

Stained samples were analyzed as z-stacks (12.48 μm interval) using confocal microscope (Airyscan LSM800 Confocal Microscope, Zeiss) with the following channels: DAPI (405 nm), Alexa488 (488 nm), Texas red (561 nm), and ESID-T1 (transmitted light). For further analysis z-stacks of fluorescent channels were merged into a single plane with Zen Software (Zen 2.3 (blue edition), Carl Zeiss Microscopy).

#### 2.4.3. Image Analysis

Cell alignment was analyzed using fast Fourier transformation (FFT) and “Oval Profile” [79] plugin in ImageJ as previously described by Tognato et al. [80]. Briefly, confocal images (*n* ≥ 9) of merged channels were imported in ImageJ and an unsharp mask with radius 1 and mask 0.65 was applied. FFT was performed to display power spectrum in polar coordinates, following rotation by 90° reversing intrinsic rotation resulting from FFT. A circular selection on the power spectrum with radius width/4 was selected subsequently. “Oval Profile” plugin was executed and resulting pixel intensities were normalized for further analysis. Gray-value profiles were integrated in the region of interest (direction of printing evaluated by ESID channel ± 10°) and divided by the total area under the curve (AUC) to semi-quantify average degree of alignment.

### 2.5. ECM Production

After 4 weeks of culture, samples were fixed in 4% paraformaldehyde as described in Section 2.4.1. Cells were incubated in blocking solution containing 10% goat serum in 0.1% PBS-Tween (0.1% Tween-20 in PBS) and stained with primary antibody against collagen type I (host mouse, 1:5000 in PBS-Tween), decorin (6D6, host mouse, 37 µg/mL, DSHB (Developmental Studies Hybridoma Bank), Iowa, USA) and fibromodulin (FMOD, host rabbit, 0.5 mg/mL, Thermo Fisher Scientific). Secondary antibody AlexaFluor647 (goat anti-mouse or goat anti-rabbit IgG, 2 ng/mL, Invitrogen, Thermo Fisher Scientific) was used at 1:200 dilution in PBS-Tween. Cell nuclei were counterstained with DAPI (500 µg/mL) at a dilution of 1:500 in PBS-Tween. Control samples were stained without primary antibodies to ensure no nonspecific binding of secondary antibody. Samples were analyzed using confocal microscopy with the following channels: Alexa647 (647 nm) and DAPI (405 nm). Images were merged as described in Section 2.4.2.

### 2.6. Glycosaminoglycan and DNA Content

Cell-seeded scaffolds were collected at weeks 0 and 4 and digested in proteinase K solution (0.5 mg/mL in phosphate buffer, pH 6.5, Roche, Basel, Switzerland) for 48 h at 56 °C. DNA content was measured by using Hoechst 33,258 and absorbance at 360 nm (excitation) and 465 nm (fluorescence emission) was read. Calf thymus DNA (100 µg/mL, Invitrogen, Thermo Fisher Scientific) was used to create the DNA standard. Sulphated glycosaminoglycan (sGAG) content was determined using 1,9-dimethyl-methylene blue (16 ng/mL, pH 3). Absorbance was read at 535 nm with a Micro Plate Reader (Tecan, Maennedorf, Switzerland). sGAG concentration was calculated from a standard curve obtained with chondroitin 4-sulfate sodium salt from bovine trachea (1 mg/mL) and normalized to DNA content.

### 2.7. Gene Expression

RNA was isolated from monolayer (day 0) and week 4 scaffolds using TRI-reagent. For each condition, nine scaffolds were collected and pooled in sets of three to generate three independent RNA samples (*n* = 3). Each pooled sample was homogenized with a Mixer Mill (MM400, Retsch GmbH, Haan, Germany). TaqMan^TM^ Reverse Transcription Kit was used to generate cDNA from a total 0.5 µg RNA per sample. Relative gene expression reactions were set up in 10 µL reaction mixes using TaqMan^TM^ MasterMix, relevant human primers (Supplementary Table S1), diethyl pyrocarbonate-treated water (DEPC-water) and cDNA. Real-time polymerase chain reaction (real-time PCR) was performed on duplicates using QuantStudio 7 Flex (Applied Biosystems, Thermo Fisher Scientific, Waltham, MA, USA). The relative gene expression was identified using 2^−ΔΔCt^ value [81,82] with RPLP0 as endogenous control and week 0 samples were used for normalization.

### 2.8. Statistical Analysis

Quantitative data were analyzed using GraphPad Prism (version 8.1.0). Data distributions were first assessed for normality. Normally distributed datasets were analyzed using one-way ANOVA followed by Tukey’s multiple-comparisons test, while non-normally distributed data were analyzed using the Kruskal–Wallis test with Dunn’s post hoc correction.

In addition to hypothesis testing, effect sizes (Cohen’s d) and 95% confidence intervals were calculated for pairwise comparisons to quantify the magnitude and uncertainty of alignment differences between scaffold geometries. Data are presented as boxplots showing the median and interquartile range, with summary statistics additionally reported as mean ± standard deviation. A *p*-value < 0.05 was considered statistically significant.

## 3. Results

### 3.1. Material Characterization of 3D-Printed Constructs

#### 3.1.1. Light Microscopy

**Figure 1:**
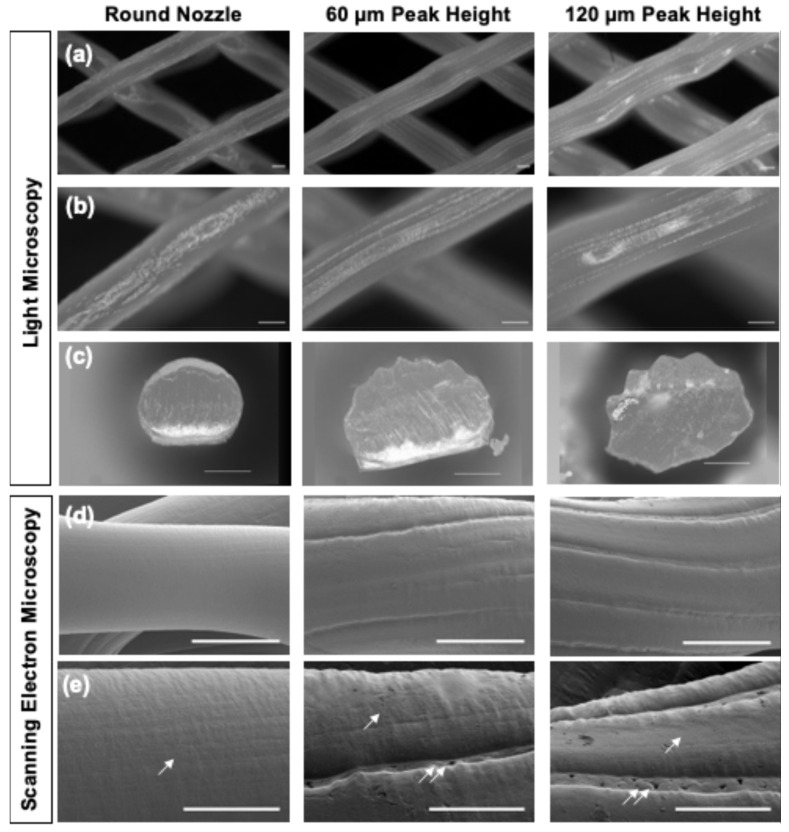
Image-based characterization of PCL scaffolds printed using different nozzle geometries (round, 60 µm peak height, and 120 µm peak height). (**a**,**b**) Representative low-magnification (2.5×) light microscopy top-view images of printed struts as fabricated, providing an overview of filament geometry and surface patterning. (Scale bars: 200 µm.) (**c**) Cross-sectional profiles of individual printed filaments. (Scale bars: 200 µm.). (**d**,**e**) Scanning electron microscopy (SEM) top-view images of strut surfaces at increasing magnification. (Scale bars: 300 µm in (**d**) and 100 µm in (**e**)). Across all nozzle designs, extruded filaments exhibited aligned surface textures (single arrows), while extrusion through patterned nozzles additionally produced uniaxially aligned concave microgrooves (double arrows).

Custom-designed printer nozzles with periodic circumferential patterns of different peak sizes were used to induce aligned surface topography on the 3D-printed constructs. Microscopic imaging of extruded filaments revealed that the spaces between the peaks in the nozzle shape resulted in uniaxially aligned surface grooves (Figure 1a,b). Cross-sectional images demonstrated extruded filaments possessing circumferential peaks with average height of 17.87 ± 5.69 µm and 31.46 ± 8.09 µm for the nozzles with 60 and 120 µm peak height, respectively (Figure 1c).

#### 3.1.2. Scanning Electron Microscopy

Scanning electron microscopy (Figure 1d,e) revealed that all nozzles, independent of their shape, produced filaments with slight aligned textures across the surfaces (single arrows in Figure 1e), attributed to inherent stretching of the polymer melt post-deposition due to nozzle translation. Extrusion through the patterned nozzles additionally generated distinct uniaxial aligned grooves (double arrows in Figure 1e) along the printed filaments that were 10.87 ± 3.09 µm or 17.77 ± 4.91 µm wide for the nozzle with 60 or 120 µm peak height, respectively. Furthermore, filament diameter was calculated to be 388.09 ± 19.20 µm for the round and 583.57 ± 16.77 µm or 667.49 ± 16.03 µm for the nozzles with 60 or 120 µm peak height, respectively (Figure 2a).

#### 3.1.3. Wettability

Water contact angle measurements revealed that increasing peak height on the PCL filaments resulted in a decreased water contact angle, indicating increased surface wettability [83]. This reduction in contact angle is attributed to the formation of more complex microscale surface features, specifically concave grooves, which may increase surface roughness and promote liquid spreading via roughness-induced wetting effects [84]. Accordingly, filaments extruded through patterned nozzles exhibited significantly lower contact angles (*p* < 0.05) compared to those extruded through the round nozzle. The average contact angles were 65.09 ± 2.72° for round nozzles and 54.94 ± 5.31° or 56.21 ± 2.13° for the 60 µm and 120 µm patterned nozzles, respectively (Figure 2c).

To facilitate comparison across nozzle groups, key architectural descriptors are summarized in Table 1 below. The patterned nozzles increased filament diameter and layer thickness (0.3 mm for round vs. 0.5 mm for patterned nozzles) to accommodate circumferential peaks, while also reducing water contact angle (indicating improved wettability due to increased surface roughness).

### 3.2. Cell Alignment and Proliferation on the 3D-Printed Scaffolds

**Figure 3:**
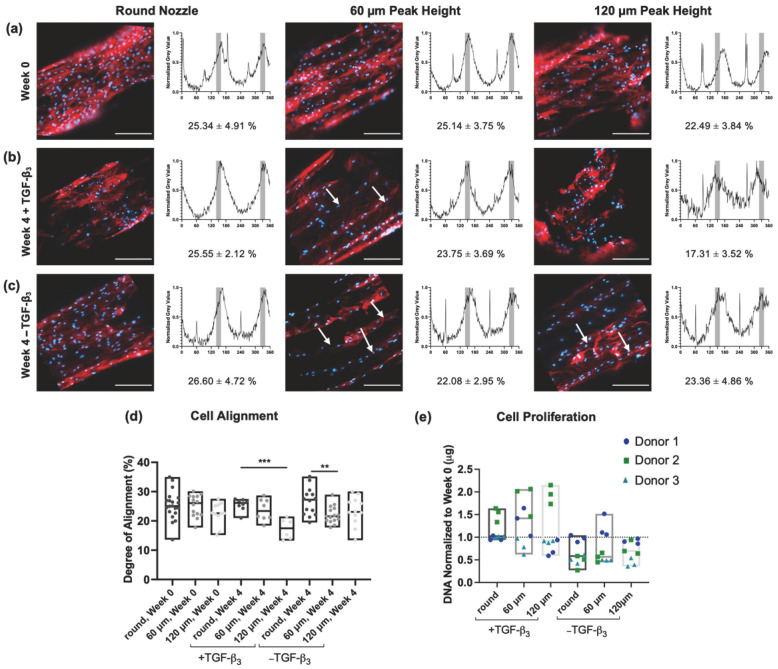
BM-MSCs on PCL scaffolds stained with phalloidin (red) and DAPI (blue) at week 0 (**a**) and after 4 weeks of culture in medium with (**b**) or without TGF-β_3_ (indicated as −TGF-β_3_) (**c**) with gray-value profile resulting from FFT. White arrows indicate the printing direction and highlight representative examples of cells confined and aligned end-to-end within the concave microgrooves on patterned-nozzle scaffolds. Degree of alignment was calculated by the portion of AUC at the region of interest (printing direction ± 10°, highlighted in gray). (Scale bars: 200 µm). (**d**) Statistical analysis of degree of alignment revealed a significant difference between the indicated groups (*** *p* < 0.001, ** *p* < 0.01). Effect sizes (Cohen’s d) with 95% confidence intervals for mean differences provided in Supplementary Table S2. (**e**) DNA content at week 4 normalized to week 0 (donor 1 dark blue circles, donor 2 green squares, donor 3 cyan triangles).

BM-MSCs cultured on the 3D-printed scaffolds were stained with anti-vinculin, phalloidin and DAPI, and imaged using confocal microscopy. Representative images of cell-seeded scaffolds printed with each nozzle geometry, corresponding gray-value profiles obtained from FFT analysis, and calculated degrees of alignment are shown in Figure 3a–c.

At week 0, cellular alignment was comparable across all nozzle geometries. Cells seeded on scaffolds printed with the round nozzle exhibited an average alignment of 25.34 ± 4.91%, while those printed with the 60 µm peak height nozzle showed 25.14 ± 3.75%, corresponding to a negligible effect size (Cohen’s d = 0.04) and a mean difference of 0.19% (95% CI: −2.83 to 3.22; *p* = 0.90). Alignment on scaffolds printed with the 120 µm nozzle (22.50 ± 3.84%) was modestly lower than that of the round nozzle, corresponding to a moderate effect size (d = 0.62); however, this difference was not statistically significant (mean difference = 2.84%, 95% CI: −0.42 to 6.11; *p* = 0.11). One-way ANOVA confirmed no significant differences in alignment between nozzle geometries at week 0 (*p* > 0.05) (Figure 3d).

After 4 weeks of culture in differentiation medium supplemented with TGF-β3, cells seeded on scaffolds printed with the round nozzle exhibited the highest degree of alignment (25.55 ± 2.12%) (Figure 3b). Compared to the 60 µm patterned nozzle (23.75 ± 3.69%), this corresponded to a moderate effect size (d = 0.60) but did not reach statistical significance (mean difference = 1.79%, 95% CI: −1.43 to 5.02; *p* = 0.25). In contrast, alignment on scaffolds printed with the 120 µm nozzle (17.31 ± 3.52%) was markedly lower than that of the round nozzle, corresponding to a very large effect size (d = 2.95) and a significant mean difference of 8.24% (95% CI: 4.71 to 11.76; *p* < 0.001) (Figure 3d).

Under control conditions (without TGF-β3), cells cultured for 4 weeks on scaffolds printed with the round nozzle exhibited the highest degree of alignment (26.60 ± 4.72%), followed by those printed with the 60 µm (22.08 ± 2.95%) and 120 µm (23.36 ± 4.86%) patterned nozzles (Figure 3c). Alignment on round-nozzle scaffolds was significantly greater than that on 60 µm patterned scaffolds, with a large effect size (d = 1.05) and a mean difference of 4.20% (95% CI: 1.19 to 7.20; *p* < 0.01). The difference between round and 120 µm nozzles corresponded to a moderate effect size (d = 0.61) but was not statistically significant (mean difference = 2.93%, 95% CI: −0.89 to 6.76; *p* = 0.13).

From week 0 to week 4 under control conditions, alignment values remained largely unchanged for round and 60 µm nozzles, while a slight increase was observed for 120 µm scaffolds (*p* > 0.05) (Figure 3d). At week 4, TGF-β3 supplementation produced geometry-dependent effects, with the largest change in alignment being a decrease observed for the 120 µm nozzle geometry, but changes were not significant (*p* > 0.05).

Wang and colleagues analyzed cellular alignment over 180° and concluded that a degree of cellular alignment greater than 11.7% is defined as anisotropic [85]. In an isotropic material, cells are equally distributed and 0.56% of the cells will be located in a region of 1°. By applying this concept into our area of interest (direction of printing ± 10°) a degree of alignment above 23.40% indicates anisotropic alignment. Using this criterion, cellular alignment values at or near the anisotropic threshold were observed across scaffolds printed with all nozzle geometries at week 0 and under control conditions. In contrast, the lowest alignment values were observed for scaffolds printed with the 120 µm patterned nozzle and cultured in the presence of TGF-β3 at week 4, and overall, alignment values in the +TGF-β3 groups tended to be lower than those in control conditions.

These differences may be interpreted in the context of cell proliferation and effective surface density, while accounting for donor-to-donor variability. DNA quantification revealed substantial inter-donor variability, particularly under +TGF-β3 conditions, consistent with known heterogeneity in primary MSC proliferative responses [86,87,88]. Despite this variability, a consistent trend was observed across donors: relative to week 0, scaffolds cultured under control conditions exhibited a decreasing trend in DNA content over time (*p* > 0.05), whereas scaffolds cultured with TGF-β3 showed an increasing trend (*p* > 0.05) (Figure 3e). These observations suggest that differences in effective cell density may influence the extent to which cells respond to topographical cues. At lower surface densities, cells may more readily sense and respond to local topographical features, whereas higher densities may promote crowding and increased cell–cell interactions, reducing the relative contribution of surface topography to alignment [89,90].

FFT is a global metric that does not capture the distinct groove-confined columnar organization observed exclusively on patterned scaffolds (white arrows, Figure 3c). While scaffolds printed with the round nozzle exhibited a continuous, web-like distribution of actin and vinculin across the filament surfaces, cells on filaments printed with patterned nozzles preferentially localized within the concave grooves, organizing end-to-end into longitudinal rows that were often one cell wide for the 60 µm peak-height nozzle and two cells wide for the 120 µm peak-height nozzle. Although no statistically significant differences in overall alignment were observed among nozzle types, qualitative differences in cellular organization were apparent between round and patterned scaffolds, indicating that surface topography influenced spatial distribution rather than the magnitude of cell alignment.

### 3.3. ECM Production

**Figure 4:**
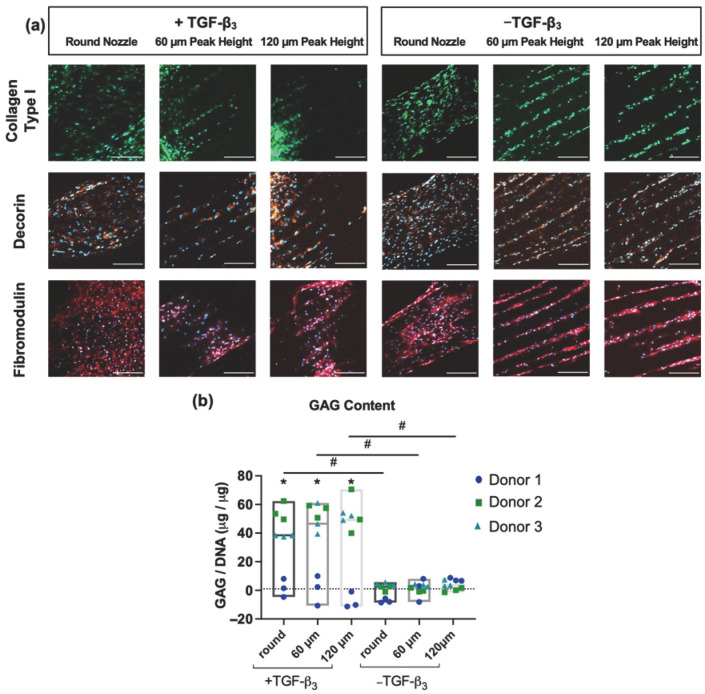
(**a**) Representative images of collagen type I (green), decorin (orange), fibromodulin (red), and nuclei (DAPI, blue) in BM-MSCs (all donors) cultured on PCL scaffolds for 4 weeks in medium with (+TGF-β_3_) or without (−TGF-β_3_). The microgroove patterning was more faithfully retained in the absence of TGF-β_3_, where ECM deposition occurred preferentially within the concave microgrooves as organized columnar arrays. (Scale bars: 200 µm). (**b**) Quantitative measurement of sGAG per DNA on the scaffolds indicated higher sGAG production for the group cultured with TGF-β_3_ in the medium. (Donor 1 dark blue circles, donor 2 green squares, donor 3 cyan triangles). * indicates *p* < 0.05 versus week 0, # indicates *p* < 0.05 between groups.

Confocal immunofluorescence was used for qualitative evaluation of ECM production on the scaffolds at 4 weeks of culture. Deposition of collagen I, the major ECM component of the outer AF, and small leucine-rich proteoglycans (SLRPs), decorin and fibromodulin, corresponded with cellular organization on the scaffolds (Figure 4a). Notably, patterning effects were more consistently maintained without TGF-β3 supplementation, and ECM deposition was localized specifically within the concave microgrooves, forming columnar arrays. The presence of sGAG on the scaffolds was verified quantitatively with a 1,9-dimethyl-methylene blue assay. Total sGAG, when normalized to DNA content on the scaffolds, increased among all nozzles compared to week 0; however, one-way ANOVA revealed that the increase was statistically significant only for the +TGF-β_3_ group (*p* < 0.05). Additionally, the +TGF-β_3_ group produced significantly more (*p* < 0.05) sGAG compared to the −TGF-β_3_ group (Figure 4b) for all the nozzle patterns.

### 3.4. Gene Expression

**Figure 5:**
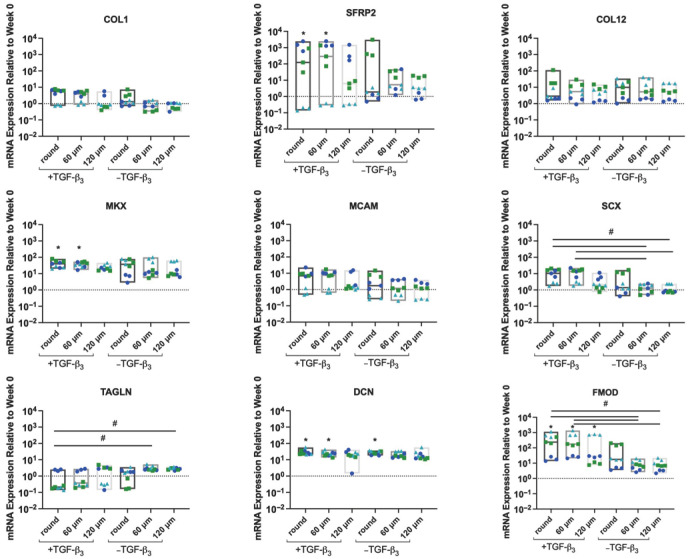
Gene expression data after 4 weeks of culture on PCL scaffolds in medium with (+TGF-β_3_) or without (−TGF-β_3_), normalized to the endogenous control and to baseline expression prior to scaffold culture (week 0). (Donor 1 dark blue circles, donor 2 green squares, donor 3 cyan triangles). * indicates *p* < 0.05 versus week 0, # indicates *p* < 0.05 between groups.

The gene expression study evaluated markers characteristic for the outer AF: collagen type I (COL1) [91], secreted frizzled-related protein 2 (SFRP2) [91], collagen type XII (COL12) [91,92], Mohawk (MKX) [93], CD146 (MCAM) [94], scleraxis (SCX) [95], and transgelin (TAGLN) [94] and markers associated with fibrillogenesis: fibromodulin (FMOD) and decorin (DCN) [92] (Figure 5). Trending upregulation was observed for all markers compared to week 0 regardless of TGF-β_3_ supplementation, with statistically significant increases (*p* < 0.05) for AF markers SFRP2 and MKX, in the +TGF-β_3_ cells cultured on scaffolds printed with the round and 60 µm nozzle peak heights. DCN followed the same trend with statistically significant increases in its expression compared to day 0 for the +TGF-β_3_ scaffolds printed with the round and 60 μm nozzle peak heights. FMOD was significantly upregulated compared to week 0 for all the nozzles in the presence of TGF-β_3_ (*p* < 0.05). FMOD and SCX exhibited a significantly higher upregulation for the +TGF-β_3_ compared to −TGF-β_3_ group. TAGLN expression was higher for the control group (without TGF-β_3_) on scaffolds printed with the patterned nozzles compared to the +TGF-β_3_ group on scaffolds printed with the round nozzle (*p* < 0.05). COL1 and SCX exhibited a trend with nozzle pattern, of increasing expression with decreasing size of the topography, yet the differences were not statistically significant (*p* > 0.05).

## 4. Discussion

This study introduces a novel single-step extrusion-based 3D-printing method for fabricating PCL AF scaffolds that incorporate aligned concave microscale topographical cues. The approach was designed to promote cell elongation and guide the formation of organized, AF-like tissue. This approach also addresses key limitations of existing scaffold fabrication methods, including the inability of standard extrusion-based printing to generate the cell-scale features necessary for directing cell alignment [61,62] and the reliance of other techniques on multistep assembly or material-specific requirements [48,96,97,98,99,100]. By addressing these challenges, this work provides a streamlined and integrative biofabrication strategy for generating aligned 3D lamellar architectures that replicate the organization of native AF tissue. The novelty of this approach lies in the use of custom-patterned nozzles to impart concave microgrooves directly during extrusion, enabling integration of microscale surface topography with multilamellar angle-ply architecture in a single fabrication step without post-processing.

Physical characterization of our 3D-printed PCL scaffolds confirmed microscale surface patterning on the extruded struts, achieved through custom-manufactured patterned nozzles. Light microscopy and scanning electron microscopy (SEM) revealed uniaxially aligned concave grooves and surface textures directly imparted by the extrusion process. Nozzles with 60 µm and 120 µm nominal peak heights produced concave grooves approximately 10 µm and 17 µm wide, respectively, running longitudinally along the strut axis.

To contextualize these concave microgrooves relative to established AF alignment strategies, it is important to consider how cellular organization has traditionally been achieved. Most tissue-engineering strategies for inducing cellular alignment rely on electrospun nanofibrous mats, where cells elongate extensively along individual convex fibers [101,102,103,104], generating high cytoskeletal tension [105,106,107], nuclear deformation [108,109], and oriented ECM deposition [110] through contact guidance and curvature-mediated mechanotransductive signaling pathways [84,111]. These convex surfaces promote nuclear bending and actomyosin contractility [112,113,114,115]. In contrast, our scaffolds—fabricated using patterned nozzles—present concave microgrooves that drive exclusive end-to-end, columnar cell organization within the grooves (Figure 3 and Figure 4). Concave curvature is reported to reduce nuclear deformation relative to convex substrates [112,113], thus suggesting a distinct alignment mechanism compared to previous work with electrospun nanofibrous materials.

Notably, all nozzles produced a weakly aligned surface texture on the struts upon extrusion (Figure 1e). On struts from patterned nozzles, this weak texture appeared between microgrooves, yet cells still tended to preferentially localize within the concave grooves rather than on the intervening weakly aligned regions. In contrast, cells on round (control) nozzles interacted exclusively with the weakly aligned texture. We propose that the reduced nuclear strain on concave surfaces facilitated this preferential groove localization observed in constructs printed with patterned nozzles [112,113].

Overall, the highly ordered columnar arrays in this work recapitulate aspects of native AF cell organization—specifically the aligned chains within lamellar bundles—more closely than the continuous cell coverage observed on nanofiber mats [116,117]. Collectively, these observations position concave microgroove-guided columnar organization as a distinct architectural paradigm relative to prior nanofiber-based approaches. Table 2 provides a side-by-side comparison of relevant AF scaffold fabrication strategies, resulting topographical cues, and cellular organization in each published study, showing how our concave microgroove strategy is different from prior methods.

Given the established link between surface curvature, mechanotransduction, and lineage specification [113], we checked if the concave microgrooves generated by our patterned extrusion approach provided any differences in gene expression. We evaluated markers associated with AF tissue, AF cell differentiation, and AF phenotype, including COL1, SFRP2, COL12, MKX, MCAM, SCX, and transgelin TAGLN, and fibrillogenesis markers FMOD and DCN [93,118,119,120,121]. While microgroove topography reorganized cell position and alignment, it did not produce significant changes in AF-associated gene expression relative to week 0 across the markers assayed. This suggests that the primary effect of concave topography under these conditions was organizational rather than transcriptional. In contrast, TGF-β3 supplementation significantly increased SFRP2, MKX, DCN, and FMOD (*p* < 0.05). Because these increases were not observed on the same topographies in the absence of TGF-β3, the data indicate that growth factor exposure, rather than surface topography, was the dominant driver of the transcriptional response under our conditions.

Although concave microtopography did not independently drive broad AF lineage marker expression under these conditions, MSC expression of the actin-binding protein TAGLN—a marker of myosin II-dependent cytoskeletal tension [122]—was significantly elevated (*p* < 0.05) on 60 µm and 120 µm patterned scaffolds under control conditions compared to the +TGF-β3 condition on round-nozzle scaffolds. Notably, this increase in TAGLN expression occurred under conditions where groove-confined, columnar cellular organization was most clearly maintained, suggesting that spatial confinement imposed by concave microgrooves may be associated with altered cytoskeletal tension.

This aligns with our previous tendon-focused study using the same biofabrication platform, where the 60 µm pattern similarly enhanced longitudinal organization and upregulated the tension-responsive transcription factor SCX in engineered MSCs [67]. As both TAGLN and SCX are linked to cytoskeletal tension pathways [123,124,125], these collective findings suggest that microgroove-guided columnar alignment may promote contexts involving shared axial force transmission, coinciding with upregulation of mechanotransduction-related markers. Future work incorporating additional tension-sensitive markers, contractile inhibitors, or traction force microscopy will be required to directly test this hypothesis.

Although macro-architecture was held constant across groups using an identical ±30° angle-ply lattice, the patterned nozzles increased filament diameter (~584–667 µm vs. ~388 µm for round nozzles) and layer thickness (0.5 mm vs. 0.3 mm). We therefore considered whether these differences could influence cell seeding density, nutrient and oxygen diffusion distances, or local crowding during proliferation. However, with growth factor conditions held constant, DNA content was similar across the round, 60 µm, and 120 µm patterned groups despite these structural differences (Figure 3e). Scaffold structure was dominated by large inter-strut voids inherent to the open grid design (Figure 1a), indicating comparable macro-porosity across groups. These findings indicate that microscale concave topography, rather than macro-architectural variation, was the primary driver of cellular organization, consistent with our prior tendon study using the same platform [67].

From a translational perspective, prior implantation studies using stacked electrospun sheets [126] and melt-extruded PCL scaffolds [127] provide valuable precedent for AF repair after discectomy. Thanks to the material’s ductility and fracture toughness [128,129], PCL scaffolds could be cut intraoperatively to fit defects and implanted with adjunct containment strategies (e.g., polymer patches) to prevent extrusion. Together, these studies support the use of PCL constructs as surgically adaptable repair materials within standard operative workflows.

Our single-step extrusion-based printing approach offers potential advantages by directly incorporating cell-instructive topographical cues during fabrication, avoiding both the coarser architectures of melt extrusion [59,60] and the multistep assembly required for electrospun constructs [37,48,49,50,51,52,53]. Importantly, in our prior in vivo study with tendon repair, constructs printed through patterned nozzles were successfully sutured, implanted, and—when combined with delivered cells—supported superior tissue organization and biomechanical performance compared to acellular controls [67]. These findings demonstrate the feasibility of surgical handling and suggest the potential benefit of incorporating cells to enhance regenerative outcomes. Although our study only examined scaffold architecture and cell–material interactions in vitro, future in vivo work will be needed to evaluate fixation, retention, handling, and biological integration of cell-seeded constructs within the mechanically demanding AF environment under physiologic spinal loading.

While this method streamlines fabrication of aligned 3D scaffolds, several considerations remain. The primary limitation of the current study lies in feature scale; the concave microgrooves (~10–17 µm wide) are cell-sized and lack nanoscale textures within the channels. Although these microscale cues effectively guided MSC grouping, the cytoskeletons did not exhibit pronounced elongation in morphology. Incorporating nanoscale features within the concave grooves could enhance focal-adhesion signaling and strengthen the elongation-driven phenotype. Groove fidelity also depends on the rate of post-deposition cooling and may vary with temperature or print speed, affecting reproducibility if not carefully controlled. Despite these considerations, the single-step patterned-nozzle strategy provides a novel, foundational platform for generating microgrooved, angle-ply scaffolds that induce controlled cellular organization—supporting future in vitro mechanobiological studies and regenerative strategies.

## 5. Conclusions

This study introduces a single-step extrusion-based biofabrication method that generates both multilamellar, angle-ply architecture and uniaxially aligned microscale grooves through the use of patterned nozzles. These grooves guided BM-MSCs to organize in longitudinal, end-to-end rows within concave surface features—an anisotropic pattern distinct from the elongation-driven responses seen in electrospun scaffolds. Although groove-guided spatial organization did not independently increase AF-associated gene expression, it was accompanied by elevated TAGLN expression under −TGF-β3 conditions, suggesting an association between longitudinal arrangement and cytoskeletal tension that warrants further investigation.

A key advantage of this platform is its ability to produce aligned microfeatures and bulk lamellar structure simultaneously, offering a scalable and fabrication-efficient alternative to existing multi-stage methods. This work establishes a foundation for producing clinically practical AF scaffolds and provides a framework for probing how cell spatial alignment contributes to mechanobiological regulation in the AF and other aligned fibrous tissues.

## Figures and Tables

**Table 1:** Physical and surface characteristics of extruded PCL filaments produced using round and patterned nozzles (60 µm and 120 µm feature sizes). Values represent mean ± standard deviation with sample sizes indicated.

Parameter	Round Nozzle	60 µm Patterned Nozzle	120 µm Patterned Nozzle
Filament diameter (µm)	388.09 ± 19.20 (*n* ≥ 15)	583.57 ± 16.77 (*n* ≥ 15)	667.49 ± 16.03 (*n* ≥ 15)
Peak height (µm)	N/A	17.87 ± 5.69 (*n* ≥ 29)	31.46 ± 8.09 (*n* ≥ 29)
Groove width (µm)	N/A	10.87 ± 3.09 (*n* ≥ 15)	17.77 ± 4.91 (*n* ≥ 15)
Layer thickness (mm)	0.3	0.5	0.5
Water contact angle (°)	65.09 ± 2.72 (*n* ≥ 18)	54.94 ± 5.31 (*n* ≥ 18)	56.21 ± 2.13 (*n* ≥ 18)

**Table 2:** Comparison of biofabricated scaffolds for annulus fibrosus (AF) tissue engineering, highlighting methods of manufacture, topographical cues, cellular organization, and associated biological outcomes.

Study	Method of Manufacture	Topographical Cue Scale	Mode of Assembly	Cell Source and Culture Regime	Resulting Cellular Organization	Biological Outcomes
Nerurkar et al., 2009 [38]	Electrospinning of aligned PCL nanofibers	Nanoscale fiber alignment (~100 s nm)	Manual stacking and coupling of bilayers at ±30°	Bovine MSCs; fibrocartilaginous differentiation medium	Continuous over nanofibrous sheets; highly elongated and aligned along fiber axis	Strong collagen alignment; organized ECM deposition; mechanical parity with native AF after extended culture
Shamsah et al., 2020 [37]	Electrospinning PCL:PLLA nanofibers	Nanoscale fiber alignment; bilayer architecture at ±30°	Automated rolling of bilayer sheets into multilayered rings	Bovine AF cells; serum-supplemented DMEM with ascorbic acid	Continuous over nanofibrous sheets; elongated and directionally aligned	Directed cell orientation; aligned collagen type I deposition; improved tensile properties over culture time
Bhunia et al., 2021 [39]	Micro-extrusion 3D printing (silk–carrageenan ink)	Filament-scale architecture (~500 µm); angle-ply organization	Layer-by-layer deposition with alternating filament orientation	Porcine AF cells and ADSCs; chondrogenic differentiation medium	Continuous over smooth printed filaments; limited single-cell alignment	ECM deposition and AF-related gene expression with chondrogenic medium
Christiani et al., 2019 [56]	Extrusion-based 3D printing of PCL followed by post-printing manual surface etching	Submicron-to-few-micron grooves and ridges superimposed on strut-scale architecture	Layer-by-layer printing with secondary surface modification	Bovine AF cells; basal DMEM-based maintenance medium	Continuous over textured struts; elongated and oriented along microgrooves	Topography-driven cellular alignment; aligned ECM deposition compared to smooth (non-etched) surfaces
Present study	Single-step extrusion-based 3D printing using custom-patterned nozzles (PCL)	Concave microscale surface grooves (∼10–17 µm wide) integrated along printed filaments	Direct layer-by-layer printing of multilamellar angle-ply constructs (±30°) without post-processing	Human BM-MSCs; AF differentiation medium ± TGF-β3	Cells preferentially localized within concave grooves, forming longitudinal, end-to-end columnar arrays; limited global elongation	ECM deposition consistent with topography/cellular organization; upregulated AF-related gene expression with TGF-β3

## Data Availability

The original contributions presented in this study are included in the article/Supplementary Material. Further inquiries can be directed to the corresponding author.

## Supplementary Materials

The following supporting information can be downloaded at: https://www.mdpi.com/article/10.3390/jfb17030140/s1, Table S1: Human Primer Sequences for real-time PCR; Table S2: Pairwise comparisons of alignment values (mean differences in %, Cohen’s d effect sizes, 95% confidence intervals, and *p*-values) between round, 60 µm, and 120 µm nozzle geometries at week 0 and week 4 under control (without TGF-β3) and TGF-β3-supplemented conditions. Significant differences (*p* < 0.05) are highlighted in bold.
