# Supplementary material for: Single-Step Extrusion Printing of Microgrooved Annulus Fibrosus Scaffolds via Patterned Nozzles
Source: J Funct Biomater. 2026 Mar 11;17(3):140. doi: 10.3390/jfb17030140 (PMC13027382; doi:10.3390/jfb17030140)
Supplement: Supplementary file 1 [file jfb-17-00140-s001.zip › jfb-4089889-supplementary.pdf]

Supplementary Information for Materials and Methods

Fourier Transform Infrared Spectroscopy

PCL filaments extruded through nozzles with different peak heights were analysed by Fourier transformed infrared spectroscopy (FTIR). The measurements were performed with an attenuated total reflectance-FTIR spectroscope (Burge IR Tensor 27 Version 6.5 Build: 6.5.97) at a wavelength of 400-4000 cm^{-1}. Characteristic peaks were marked and area under the curve was calculated with OPUS Spectroscopic software (Version 6.5 Build: 6.5.97, Bruker Optik GmbH, Ettlingen, Germany).

Gene Expression Analysis

Supplementary Table S1: *Human Primer Sequences for real-time PCR*

Primer	Sequence
Collagen Type I (COL1)	*Forward*: 5'-CCC TGG AAA GAA TGG AGA TGA T-3'<br>*Reverse*: 5'-ACT GAA ACC TCT GTG TCC CTT CA-3'<br>*Probe*: 5'-CGG GCA ATC CTC GAG CAC CCT -3'
Colagen Type XII (COL12)	Assay on Demand (Hs00189184_m1 COL12A1)
MCAM / CD146	Assay on Demand (Hs00174838_m1 MCAM)
Transgelin (TAGLN)	Assay on Demand (Hs00162558_m1 TAGLN)
Secreted Frizzled-Related Protein 2 (SFRP2)	Assay on Demand (Hs00293258_m1 SFRP2)
Mohawk (MKX)	Assay on Demand (Hs00543190_m1 MKX)
Scleraxis (SCX)	Assay on Demand (Hs03054634_m1 SCX)
Decorin (DCN)	Assay on Demand (Hs00370384_m1 DCN)
Fibromodulin (FMOD)	Assay on Demand (Hs05632658_s1 FMOD)
RPLP0	*Forward*: 5'-TGG GCA AGA ACA CCA TGA TG-3'<br>*Reverse*: 5'-CGG ATA TGA GGC AGC AGT TTC-3'<br>*Probe*: 5'-AGG GCA CCT GGA AAA CAA CCC AGC-3'

Supplementary Table S2. *Pairwise comparisons of alignment values (mean differences in %, Cohen's d effect sizes, 95% confidence intervals, and p-values) between round, 60 µm, and 120 µm nozzle geometries at week 0 and week 4 under control (without TGF-β3) and TGF-β3-supplemented conditions. Significant differences (p < 0.05) are highlighted in bold.*

Comparison	Cohen's d	Mean Difference (%)	95% CI	p-value
Week 0: Round vs 60 µm	0.04	0.19	−2.83 to 3.22	0.9
Week 0: Round vs 120 µm	0.62	2.84	−0.42 to 6.11	0.11
Week 4 +TGF: Round vs 60 µm	0.6	1.79	−1.43 to 5.02	0.25
Week 4 +TGF: Round vs 120 µm	**2.95**	**8.24**	**4.71 to 11.76**	**<0.001**
Week 4 Ctrl: Round vs 60 µm	**1.05**	**4.2**	**1.19 to 7.20**	**<0.01**
Week 4 Ctrl: Round vs 120 µm	0.61	2.93	−0.89 to 6.76	0.13

Supplementary Figures

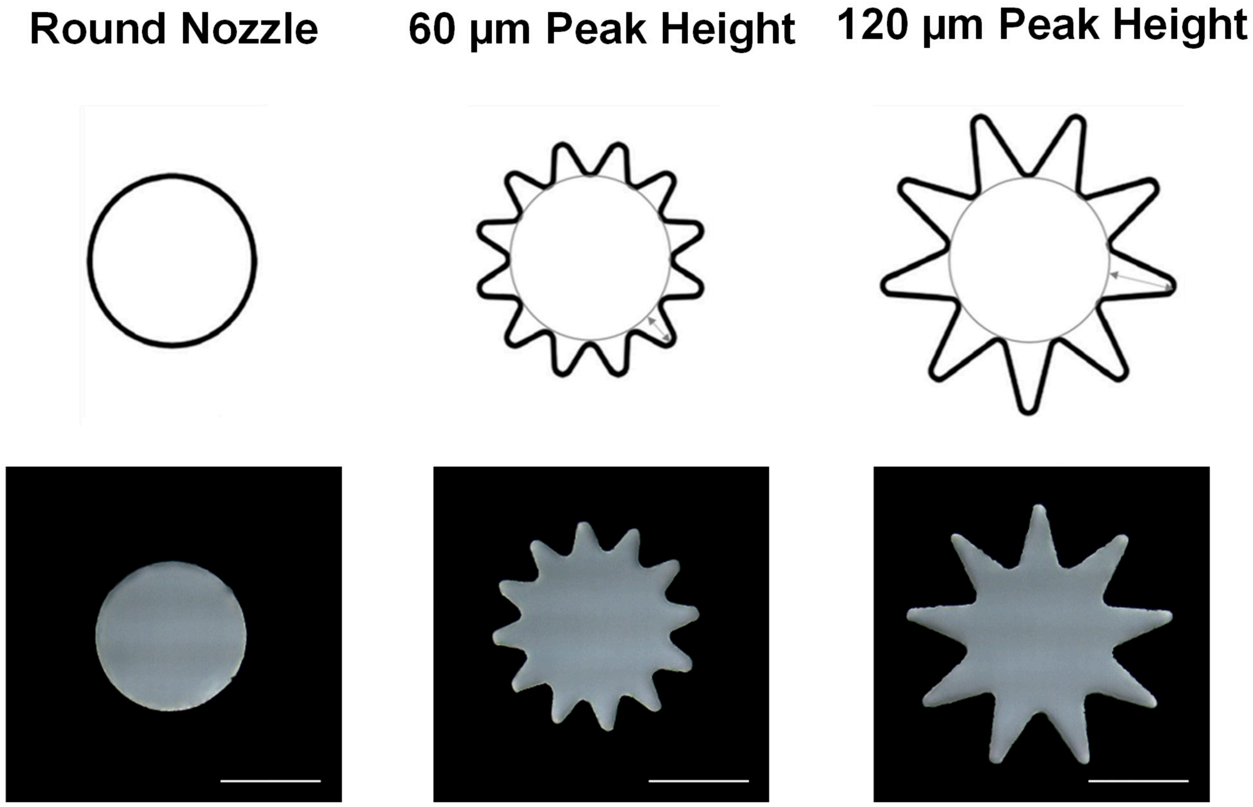

Supplementary Figure S1: (a) *Schematics of nozzle geometry and (b) light microscopy images of the engineered 3D printing nozzles with inner diameter of 300 µm and circumferential sinusoidal patterns with 60 and 120 µm peak height (Scalebars 200 µm).*

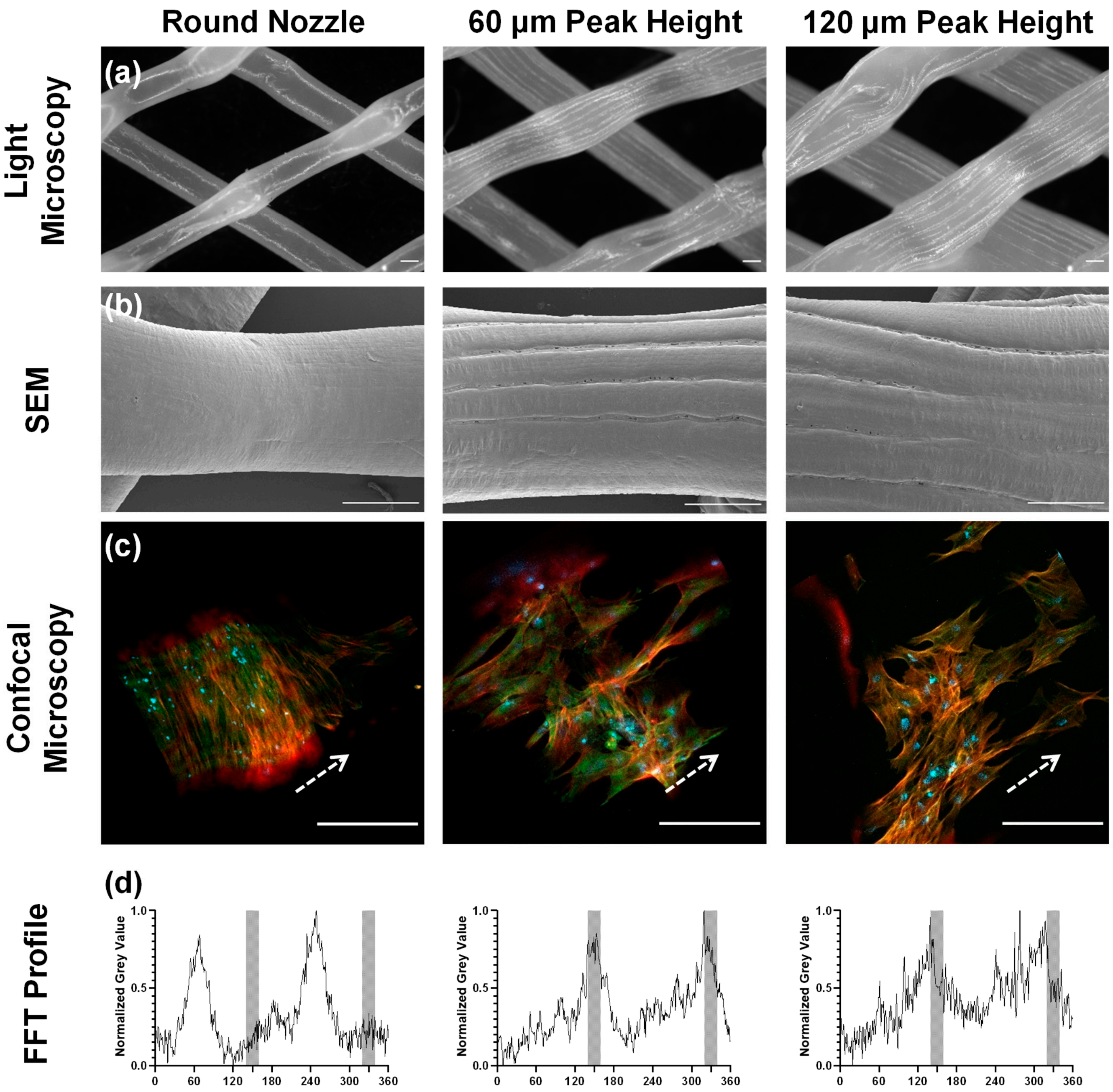

Supplementary Figure S2: *Printing with 8 mm/s resulted in sagging of the filaments interfering topographical guidance.* ***(a)*** *Light microscopy top-view images of the uniaxial aligned surface topographies printed with 8 mm/s.* ***(b)*** *Scanning electron microscopy top-view images of the surface topographies.* ***(c)*** *BM-MSCs on PCL scaffolds stained with anti-vinculin (green), phalloidin (red) and DAPI (blue) after 4 weeks culturing in medium with TGF-β_3. The white arrows indicate the direction of printing.* ***(d)*** *Grey value profile resulting from FFT. (Scalebars 200 µm)*

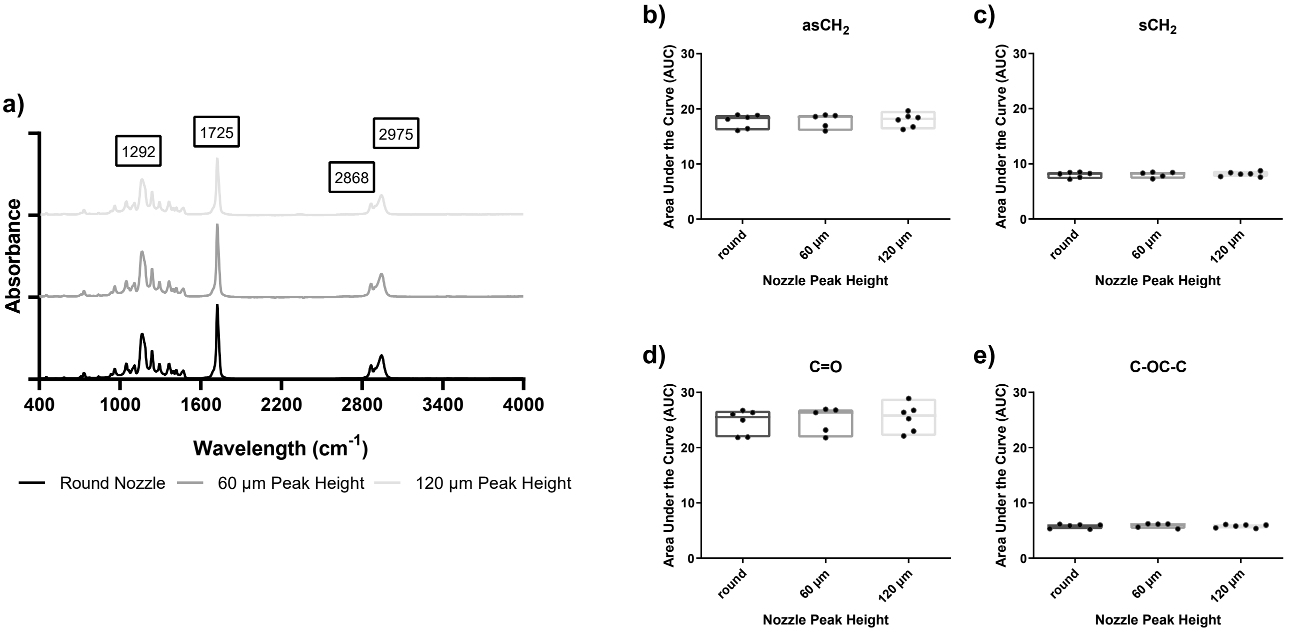

Supplementary Figure S3: *Fourier-transform infrared spectroscopy (FTIR) of PCL filaments revealed characteristic signal peaks. The typical signal peaks with their wavelengths are highlighted* ***(a)****. FTIR indicated no differences in the area under the curve as a function of nozzle geometry for characteristical bands: asymmetric* ***(b)*** *and symmetric* ***(c)*** *CH_2 (2975 and 2868 cm^{-1}), C-O backbone (1292 cm^{-1})* ***(d)*** *and carbonyl stretching (1725 cm^{-1})* ***(e)****.*
